# Pathogenic Mechanisms of the Severe Acute Respiratory Syndrome Coronavirus 2 and Potential Direct and Indirect Counteractions by Intermittent Fasting

**DOI:** 10.3390/nu15010020

**Published:** 2022-12-21

**Authors:** Benjamin D. Horne, Thomas Bunker

**Affiliations:** 1 Intermountain Medical Center Heart Institute, Salt Lake City, UT 84107, USA; 2 Division of Cardiovascular Medicine, Stanford University, Stanford, CA 94305, USA; 3 Independent Researcher, Boulder, CO 80301, USA

**Keywords:** COVID-19, intermittent fasting, periodic fasting, SARS-CoV-2, complementary therapy

## Abstract

The coronavirus disease 2019 (COVID-19) pandemic created an unprecedented burden on human health and on the function and interaction of societies across the globe. Public health preventive measures, vaccines, and antivirals were key components of the world-wide response to the health emergency. Due to the uncoordinated and variably successful response to COVID-19 and the ability of the severe acute respiratory syndrome coronavirus 2 (SARS-CoV-2) to rapidly mutate, SARS-CoV-2 continues to create considerable difficulty for humanity today. Additional preventive or therapeutic modalities are needed to help people to achieve the best possible health outcomes in the context of the evolving COVID-19 threat. Intermittent fasting is a potential complementary therapy that not only impacts chronic disease risk but also has good evidence of an impact on infectious diseases. While the data regarding fasting and COVID-19 outcomes are very limited, the conceptual connection of fasting to better outcomes includes a variety of mechanisms in human biology. This paper reviews the known mechanisms of disease impacted by SARS-CoV-2 infection and the potential or likely direct or indirect counteractions that fasting may provide that may reduce the severity of COVID-19 and help to realize the best possible health outcomes. Furthermore, fasting adds no financial cost to a care plan and, when practiced safely, is available to most adults without limitation. Further research is needed on the impact of intermittent fasting on human health in the fight against infectious diseases including COVID-19.

## 1. Introduction

Mark Twain, an astute observer, noted in an 1866 story that he, “has a surprise in it for those dull people who think that nothing but medicines and doctors can cure the sick. A little starvation can really do more for the average sick man than can the best medicines and the best doctors. I do not mean a restricted diet, I mean *total abstention from food for one or two days*. I speak from experience; starvation has been my cold and fever doctor for fifteen years, and has accomplished a cure in all instances” [1]. More than 150 years later, the best medicines and doctors are much more advanced, including that we have access to many more safe and effective treatments, including vaccines, antivirals, and antibiotics. Despite these important advancements, intermittent fasting (or “starvation” as Twain called it) may become a complementary therapy (note that fasting does not result in the break-down of vital organs like real starvation does). Importantly, fasting is available for free to all people regardless of location, ancestry, income, or education. Widespread individual experimentation with intermittent fasting began about 2018 when the practice moved from diet and nutrition circles into the consciousness of the general public. While the proportion of people throughout the world who have tried some form of health-related fasting is unclear, it continues to be a popular topic in the lay press, in health and research communities, and in friendly conversations.

With a substantial academic literature building up around it, fasting may be presumed to be popular due to more than fad diets and perceived results. It appears that the observed health benefits outweigh the costs and burdens of fasting. The potential health benefits of fasting are multiple, with the primary documented benefits being reductions in weight and in cardiometabolic and cardiovascular risks based on the results of randomized controlled trials [2,3,4,5,6,7,8]. Fasting can lead to weight loss, but does so only to an extent similar to the weight loss from daily calorie restriction [2,3,4,5]. Improved longevity and healthy aging are also among the expected benefits of fasting, but only observational studies of these outcomes are reported [9,10].

Conceptually, the benefits of fasting occur due to the triggering by fasting of molecular, cellular, and tissue-level mechanisms that are encoded in human DNA. These mechanisms are anticipated to have accumulated widely in the collective human genetic code historically as fasting preserved the lives of distant ancestors who were sufficiently fit to resist conditions in which a combination of low food availability and other environmental factors such as infectious diseases were present. The effect of the adverse conditions had to be just enough to induce hormesis for some individuals—a situation in which protective physiologies are triggered at low to moderate dose of fasting, while other people deficient in the protective mechanisms triggered by fasting did not have a sufficient shield from the environmental conditions and died without producing offspring. The adverse conditions could not be of a high dose that caused a profound impact in which all people experienced death or a reproduction-inhibiting harm.

Because of the lack of general knowledge prior to the twentieth century regarding prevention and treatment of infectious diseases, intrinsic protective genetic mechanisms related to fasting developed over the millennia to protect against pathogens. Such a survival benefit of intermittent fasting would logically connect to protection against infectious diseases in societies devoid of a germ theory of communicable diseases, but unfortunately the evaluation of the effect of fasting on infectious disease outcomes exists in the shadows today behind the examination of outcomes related to chronic diseases. Even more, the reduction of weight and fat mass have taken center stage in most human studies of intermittent fasting, in part due to the potential cosmetic effects of weight loss. The accumulation of cardiovascular and metabolic benefits from fasting through weight loss are also popular extensions of those investigations, while a more limited set of human research focuses on weight loss-independent cardiometabolic effects [6,8,11].

Fasting has been known for decades to have profound effects on physiological systems related to the metabolism, heart, and kidney [12]. Nutritional regimens that include frequent fasting have been shown to reduce weight and fat mass [13]. The intense fasting diets that are geared toward rapid weight loss include twice-weekly fasting and alternate-day fasting in which energy intake is restricted during a 24-hour period on multiple non-consecutive or consecutive days each week. They also include time-restricted eating (TRE) in which energy intake only occurs during a 6–8 h period each day of the week, including early TRE where food is consumed from about 7 am until 1–3 pm and late TRE (e.g., Ramadan) where food is consumed only during the evening or nighttime. It is well-established that weight loss leads to improvement in cardiovascular risk factors and decreased risk of insulin resistance and metabolic risk factors, thus these intense fasting diets that lead to substantial rapid weight loss are also associated with reduction in cardiometabolic risk factors [13].

Uniquely, fasting reduces cardiometabolic risks even when little to no weight loss is realized [6,8,11]. This intriguing capability includes metabolic improvements that are observed when lower-frequency intermittent fasting is utilized, such as a regimen of once-per-week 24-hour water-only fasting from which substantial weight loss did not occur [8]. Very low-frequency once-per-month periodic fasting has also been studied in observational analyses for health outcomes of fasting conducted primarily for religious purposes [9,10,14]. Such very low-frequency fasting is unlikely to result in substantial weight loss, and observational studies showed that people who reported to routinely engage in such periodic fasting had similar weight and body mass index to those who did not fast routinely [9,10]. Many people (35–40%) in those studies who engaged in periodic fasting for religious purposes had participated in the behavior for >40 years (or about 67% of their lifespan) [9,10,14], thereby potentially training their bodies to respond optimally to any length of fasting. Associations of periodic fasting were found with lower risks of coronary heart disease and diabetes in cross-sectional observational analyses [9]. In prospective longitudinal observational cohort studies, periodic fasting was also associated with lower incidence of heart failure [10], and with greater longevity [10].

With the occurrence of the coronavirus disease 2019 (COVID-19) pandemic, periodic fasting was found to be associated with lower risk of hospitalization or mortality after the diagnosis of COVID-19 [14]. In a population of 1524 patients undergoing evaluation by coronary angiography for the presence of coronary artery disease during February 2013 to March 2020, 512 reported participation in routine periodic fasting for >40 years (average age was 63 years) and 1012 did not fast routinely, while 1319 tested negative for SARS-CoV-2 at community test facilities after the onset of the pandemic and 205 tested positive [14]. Periodic fasting was not associated with infection by SARS-CoV-2 (14.3% of fasters versus 13.0% of non-fasters were positive, odds ratio: 1.11, *p* = 0.51) [14]. Among those who tested positive, though, people who engaged in periodic fasting had a lower risk of the composite event of hospitalization or mortality after their COVID-19 test (HR = 0.61, 95% confidence interval 0.42, 0.90; *p* = 0.013) [14]. This association of fasting with hospitalization or mortality was not diminished by adjustment for demographics, socioeconomic parameters, physical activity or behavioral variables, cardiac risk factors, comorbidities, or medical history.

Intermittent fasting (i.e., daily or weekly patterns of energy restriction) and periodic fasting (i.e., monthly patterns of energy restriction) may strengthen the body against infectious diseases and their consequences, including COVID-19. Fasting may reduce inflammation, activate pathways that destroy pathogens, support a healthy microbiome, and trigger the innate immune system to respond to infectious disease even if the pathogen tries to shut down or evade human immunity. For example, during a fasting period while energy intake is restricted, fasting may boost physiological mechanisms of human immunity specifically related to the spike protein of the severe acute respiratory syndrome coronavirus 2 (SARS-CoV-2) [15,16], activate other mechanisms related to general human immune responses [17,18,19,20], reduce the hyperinflammatory response to SARS-CoV-2 infection [21,22,23,24,25,26], and strongly induce the cellular “housecleaning” of autophagy [19,27,28,29]. The diet and gut-microbiota play an important role in health generally [13,18], and SARS-CoV-2 can cause major adverse effects on the microbiome [30], but fasting may support a healthy microbiome and increase resistance to gut dysbiosis [16,31,32]. In the long-term, fasting may improve basal levels of factors related to the response to infectious disease and to inflammation modulation [17,23,33,34,35], and generally reduce the risk and the prevalence of morbidities that are associated with worse prognosis after COVID-19 diagnosis, such as coronary artery disease, myocardial infarction, heart failure, and diabetes [4,5,6,7,8,9,10,14,20,36,37,38,39,40].

With the continued global spread and mutation of SARS-CoV-2 and substantial numbers of people still experiencing poor outcomes of COVID-19, especially people at higher risk due to demographic, clinical, and genetic risk factors [39,40], further support for human health is needed world-wide. The evolving pandemic, although ignored in some areas, is allowing the rise of more infectious and virulent SARS-CoV-2 variants that are increasingly able to evade the human immune system and that are decreasingly responsive to therapies including vaccines and antivirals [41]. Evidence suggests that viral mutation will continue unabated for the foreseeable future [41]. The following section describes multiple mechanisms of SARS-CoV-2 pathogenic action and how they may be ameliorated by fasting. These mechanistic insights suggest a potential to use intermittent fasting in protecting against severe outcomes of acute and post-acute sequelae of COVID-19, as one observational study found [14]. Additional examinations of these proposed mechanisms in randomized controlled intervention trials of fasting are needed to directly evaluate them.

## 2. Mechanisms of SARS-CoV-2 and Potential Opposing Effects of Fasting

### 2.1. A Spike Protein Fatty Acid Binding Pocket

The SARS-CoV-2 virus is distinguished from other coronaviruses including SARS-CoV-1 in part by its unique polybasic furin cleavage site on its spike protein [15]. This aspect of the spike protein endows SARS-CoV-2 with a greater affinity for attachment and entry into human cells than other coronaviruses [15]. Further, most therapeutics for COVID-19 such as monoclonal antibody antiviral medications and the various vaccines target the spike protein to neutralize the effect of SARS-CoV-2 because the spike is specific for this virus. SARS-CoV-2 contains a fatty acid binding pocket on the pathogen’s spike protein that, when linoleic acid becomes attached to it, locks the spike protein in a conformation that is not conducive to binding the angiotensin converting enzyme 2 (ACE2) [15]. ACE2 is the primary protein that SARS-CoV-2 hijacks to infect human cells. The SARS-CoV-2 fatty acid binding pocket was recognized early in the pandemic and was determined to be a potential drug target for reducing or inhibiting the ability of SARS-CoV-2 to spread into host cells [15]. Subsequently, additional ligands were identified through functional simulations as having the potential to bind to the pocket to decrease the ability of the spike protein to adhere to ACE2, including steroids such as dexamethasone, fat-soluble vitamins including vitamins A, D, and K, and retinoids such as tretinoin, acitretin, and tazarotene [42]. This binding pocket is conserved across deadly epidemic-associated β-coronaviruses such as SARS-CoV-1, SARS-CoV-2, and the Middle East respiratory syndrome coronavirus, but is not available for binding fatty acids in endemic cold-causing coronaviruses [43]. Direct in vivo investigation of this intriguing mechanism is needed.

Notably during fasting, fatty acids including the essential fatty acid linoleic acid are increased in the circulation as the metabolic switch is thrown [16,44]. This well-established mechanism of fasting minimizes the use of glucose for energy and triggers the extraction of fatty acids from adipose cells [16,44,45]. Ketogenic diets also function by activating this switch to extract fatty acids that, in the presence of low insulin, are converted to ketone bodies such as β-hydroxybutyrate and used for energy production [45]. Both intermittent fasting and ketogenic diets may produce a benefit in the context of SARS-CoV-2 by providing elevated fatty acids that will bind to the spike protein and, to some extent, inhibit host infection. The degree to which a SARS-CoV-2 infection is minimized via this mechanism likely depends on the amount of fatty acids that are available in storage, especially since linoleic acid is an essential fatty acid that would only be available through storage capabilities during fasting. Clinical investigations are needed to study this effect.

### 2.2. T Cells and Deactivation of Ketosis

In a recent comparative study of influenza infection versus SARS-CoV-2 infection, human and mouse data revealed that infection by influenza induced ketogenesis, including an increased production of β-hydroxybutyrate [46]. In contrast, ketogenesis was impaired in moderate to severe COVID-19 and a deficit in the production of interferon-γ and other interferon-related cytokines by CD4^+^ T cells was found. However, addition of β-hydroxybutyrate to human and mouse T cells resulted in increased production of interferon-γ by CD4^+^ and CD8^+^ T cells, aided in the generation of energy and bioenergetic amino acids and in the function of mitochondria in activated T cells, and generally supported T cell survival [46]. While that was a single study and the topic is in need of further exploration, those findings reveal the potential for fasting to support the function and survival of activated T cells through the previously mentioned well-known increase of circulating fatty acids and ketones such as β-hydroxybutyrate by fasting and ketogenic diets [16,44,45], which should provide support to the antiviral immune response in people infected by SARS-CoV-2. Of note, in an observational study of people with type 2 diabetes, a medically supervised ketogenic diet was shown to be associated with a lower risk of COVID-19-related hospitalization and individuals who were not hospitalized had greater ketone levels and experienced more weight loss; levels of inflammatory factors were not tested [47].

### 2.3. Adipocyte Infection and Non-ACE2 Entry into Host Adipose Cells

Another study documented a potential non-ACE2 mechanism through which SARS-CoV-2 infects human adipocytes and adipose tissue-resident macrophages [48]. Other potential SARS-CoV-2 entry points include receptors at CD147, dipeptidyl peptidase 4, and neuropilin-1 in adipose cells [49]. While chronic inflammation is a hallmark of obesity, SARS-CoV-2 is known to cause a strong inflammatory response in adipose tissue [49]. Presumably, people with a greater amount of adipose tissue will experience substantially greater localized and systemic inflammation. This may be one reason that people with obesity are subjected to more severe COVID-19 and its associated poor health outcomes [20,37,50].

To the obverse, while a person is fasting, adipocyte contents such as fatty acids are scavenged for energy and this should lead to acutely reduced levels of inflammation in those cells [7,21,22]. Further, in the long-term, the use of repeated intermittent fasting results in the decrease of the volume of adipose tissue and a decline in levels of insulin resistance and chronic inflammation associated with obesity [4,5,8]. These acute and chronic benefits of fasting should ameliorate adiposity-related concerns of COVID-19 [20], although this requires direct testing in people with acute or post-acute COVID-19. Standard weight loss diets should also produce similar COVID-19-targeted benefits related to decreased adiposity, reduced obesity-associated inflammation, and lower the risk of poor COVID-19 outcomes, but this also requires evaluation.

### 2.4. Infection of Activated T Cells and Non-ACE2 Entry into Host T Cells

A second study that documented a potential non-ACE2 method of SARS-CoV-2 transport into host cells reported that the virus preferentially infects activated T cells [26]. The proposed mechanism for viral spread into activated T cells was through lymphocyte function-associated antigen 1 (LFA-1). Naturally, destruction of T cells by an infectious agent is a powerful action that facilitates its goal of invasive control of host resources so that the pathogen can turn those resources to its own ends, including viral replication and propagation. It may be in part that the hyperinflammation that some people experience in COVID-19 is a response to the destruction of activated T cells that results in dysregulation of the immune response as other components of the immune system attempt to compensate.

Intriguingly, several small studies of the immunomodulatory response to 24-hour or 72-hour fasting implicate a zero-calorie dietary state in blunting the CD4^+^ T cell response, deactivating a proportion of T cells, and strengthening the neutrophil response [21,22,25]. During fasting, insulin-like growth factor-binding protein 1 was increased and exerted a controlling effect that should reduce the activity of T cells in the innate immune response to infections such as SARS-CoV-2 if the host is in the fasted state [22]. Further, fasting blunted T cell activation and differentiation while reducing inflammatory cytokines and upregulating forkhead box O4 (FOXO4) [21]. Fasting-increased FOXO4 also led to downregulation of mTORC1, a protein complex central to the regulation of cellular metabolism [21]. Furthermore, a 72 h fast was found to enhance autophagy and remodel leukocyte expression with particular note of increased neutrophil and natural killer cell activity [25]. Taken together, these results suggest that intermittent fasting modifies the immune response that, in the context of COVID-19, should preferentially protect T cells from destruction by SARS-CoV-2 while strengthening other components of the antiviral immune response to eliminate this novel pathogen.

### 2.5. T Cells, the Inflammasome, and Hyperinflammation

As noted above, the CD4^+^ T cell activation of the human inflammatory cytokine cascade in response to the major histocompatibility complex binding of pathogen protein fragments (e.g., SARS-CoV-2) contributes to the characteristic hyperinflammation frequently observed in severe COVID-19 [51]. Additionally, as discussed above, during fasting the CD4^+^ T cell response is blunted and circulating inflammatory cytokines are reduced due to fasting [19,21,22,25]. More generally, NF-κB and the nod-like receptor pyrin domain 3 (NLRP3) inflammasome that are involved in the production of inflammatory cytokines through the innate immune system are also modulated by fasting, contributing to the overall ability of fasting to control the inflammatory response [19,23,24,25,52,53]. This modulating effect of fasting on inflammation was found previously in people with asthma [52], and a 7-day ketogenic diet was also previously noted to produce a similar inflammation-modulating effect [54]. Both fasting and a ketogenic diet may control and reduce the hyperinflammation of COVID-19 that can lead to respiratory failure and death [21,22,51].

### 2.6. Targeted Impairment of Autophagy

Autophagy is the selective degradation of damaged cellular components [19,27]. It results in the recycling of those components and the regeneration of healthy mitochondria, peroxisomes, ribosomes, and other cellular constituents that function effectively and efficiently. This rejuvenates cells, tissues, and the human body generally, returning it to homeostasis. Autophagy may also aid cells in adapting to new physiological conditions through the selective degradation of proteins necessary for living in the recent past and stimulation of transcription and translation of proteins composing a milieu tailored to the new environment. For example, during fasting, chaperone-mediated autophagy selectively degrades enzymes needed for glycolysis as many cells and tissues switch to burning fatty acids and ketones such as β-hydroxybutyrate and acetoacetate for energy.

Remarkably, or perhaps not so remarkably for this well-adapted virus, SARS-CoV-2 inhibits autophagy of infected human cells [55,56,57]. At least five SARS-CoV-2 proteins alter or block key steps in the autophagy pathway, including raising the pH of the lysosome and blocking autophagosome fusion with the lysosome [55,57]. This targeted corruption of human homeostasis is potentially critically damaging in shutting down a key physiological process that is especially important for the maintenance of cellular health and the reduction of oxidative stress. Because of the potential previous accumulation of oxidative damage in cells and tissues of people with certain conditions, such as obesity, diabetes, coronary artery disease, and chronic obstructive pulmonary disease, those individuals may be less able to adapt to the additional oxidative stress caused by SARS-CoV-2. Therapies that strengthen autophagy could provide important treatment effects to reduce severity of COVID-19 and a variety of anti-coronavirus pharmaceuticals are under study that appear to do just that [56].

Similarly and remarkably, fasting activates and enhances autophagy [19,27,28,29,38], potentially directly counteracting a major effect of SARS-CoV-2 infection [55,56,57]. Fasting strengthens the ability of the body to degrade damaged proteins and dysfunctional cells, including infected cells, and may thus directly trigger the destruction of a percentage of SARS-CoV-2 virions if the pathogen has gained entry into host cells. This action of fasting is non-specific, activating autophagy to the benefit of various organ systems and physiologic processes, including having effects on cognitive and metabolic pathophysiologies [28,29]. Thus, this canonical function of fasting would be expected to counteract SARS-CoV-2 even if the virus did not attempt to disable autophagy, since fasting’s trigger to enable autophagy is related to optimizing human physiological function through multiple pathways and is not solely an anti-pathogen effect [25]. Indeed, some evidence suggests that one impact of activation of autophagy is the production of ketone bodies, one of the well-recognized health effects of fasting that impacts metabolic health [58]. Another impact may be modulation of the NLRP3 inflammasome, which is more directly impactful on the innate immune system [56].

The potential benefits of fasting to stimulate the cellular “housecleaning” pathway of autophagy cannot be overstated and, while it appears that the primary effect is likely during food deprivation, further investigation is needed to elucidate whether the basal level of autophagy is increased after repeated episodes of fasting over the long-term of months to years. Anecdotal reports from individuals with post-acute sequelae of COVID-19 suggest that even 24 h of water-only fasting may induce a transient flare-up of symptoms suggestive of an increased antiviral response in a variety of tissues. Thus, studies should evaluate the variety of intermittent fasting regimens, including the daily 16–18 h fasting of TRE and 1–3 days per week of fasting, to determine if they increase autophagy during fasting or if over the long-term repeated fasting episodes increase basal levels of autophagy. Of particular note, elevated levels of autophagy increase viral antigen presentation on the surface of antigen presenting cells [59]. As increased autophagy also increases protein degradation via the ubiquitin proteasome system, both major histocompatibility complex class I and II may be involved in presenting viral peptides [60]. This function should increase the adaptive immune response and may be a key to the destruction of virally infected cells by natural killer cells and CD8^+^ cytotoxic T cells. If the so-called “Long COVID” involves subclinical or persistent residual infection, including infection that is hiding from the immune system, enhancement of autophagy could aid in exposing the simmering infection for final elimination.

### 2.7. Viral Suppression of Innate Immunity and Antigen Presentation

Analysis of SARS-CoV-2 viral proteins and their effects within cells have revealed that at least 18 of the 29 viral proteins actively block or suppress human cellular pathways that lead to the production of type I interferon and the activation of hundreds of interferon stimulated genes [61,62]. Within cells, retinoic acid-inducible gene-I (RIG-I) and melanoma differentiation-associated protein 5 (MDA5) sense viral mRNAs and utilize an adaptor protein called the mitochondrial antiviral signaling protein (MAVS) [61,62,63]. MAVS initiates a signal transduction pathway causing the cell to produce interferon-β, that is then exported and stimulates a signaling pathway in neighboring cells causing the induction of interferon stimulated genes. One of these is 2ʹ-5ʹ-oligoadenylate synthetase 1 (OAS1), whose gene product leads to the activation of RNAse L. RNAse L can directly destroy viral mRNAs in the cytoplasm or, even better, in membrane-bound viral replication compartments [64,65]. Unfortunately, new viral variants are evolving to become more resistant to the various types of interferons that human cells produce [66]. It is well known that the innate antiviral immune response is necessary to trigger adaptive immunity, thus it is possible that by suppressing innate immunity that SARS-CoV-2 can replicate to higher levels before triggering strong T and B cell responses. Indeed, this may account for asymptomatic transmission which is common with COVID-19 [67]. SARS-CoV-2 also suppresses antigen presentation in infected cells wherein a viral protein ORF6 decreases the expression of major histocompatibility complex class I on the surface of epithelial cells [57]. This likely reduces the effectiveness of antiviral immune surveillance and results in decreased immune surveillance by Cytotoxic T Cells. While speculative, because of the impact of fasting on autophagy and related pathways [19,57,68,69], it may be that early in a SARS-CoV-2 infection that some of the mechanisms mentioned above for fasting boost viral antigen presentation on cell surfaces or more quickly stimulate innate immunity to speed the development of adaptive immunity, leading to a less complicated or even asymptomatic course of infection with lower incidence of post-acute sequelae. The timing for this response would either require someone to be fasting frequently so that when they were first infected that the effects that occur during total energy restriction were present [7,16], or that routine repetitions of fasting had caused physiological adaptations over the long-term as is likely occurring in long-term periodic fasting [10,14,17]. Future investigations of fasting should evaluate both hypotheses.

### 2.8. Long-Term Modulation of Inflammation

While the important effects of fasting discussed above generally occur while someone is fasting, other long-term effects may also occur due to repeated intermittent fasting. Unsurprisingly, one of the reported effects of intermittent fasting is the modulation of the long-term basal levels of inflammation. In a randomized controlled trial of once-per-week 24 h water-only fasting [17], fasting increased galectin-3 (median change: +0.793 ng/mL, interquartile range: −0.538, 2.245) over a 6-month period of repeated fasting compared to controls (median change: −0.332 ng/mL, interquartile range: −0.992, 0.776). Galectin-3 changes were inversely correlated with declines in insulin resistance and metabolic syndrome that fasting induced [17]. Galectin-3 is a multi-role protein that was originally identified in the response to infection [33,34,35]. Galectin-3 responds directly and indirectly to infection (e.g., binds pathogens and activates the innate immune system), and reduces inflammation arising from NF-κB and the NLRP3 inflammasome [33,34,35]. Galectin-3 was also found to be involved in various other pathways, including in key capacities but complex relationships in the metabolism, heart failure, and fibrosis [17,23,70,71]. Of particular interest, it functions in a protective capacity related to type 2 diabetes onset, maintaining glucose homeostasis and having an inverse relationship with hemoglobin A1c levels [17,23]. As a widely-acting factor whose basal level is increased by repeated fasting over a period of months to years, a long-term elevation in galectin-3 may provide an adaptation that allows someone who chronically engages in fasting to potentially respond more quickly or effectively to conditions such as infection (e.g., by SARS-CoV-2) or chronic diseases that require modulation of inflammation to maintain homeostasis.

### 2.9. Gut Microbiome and Secondary Infections

**Figure 1:**
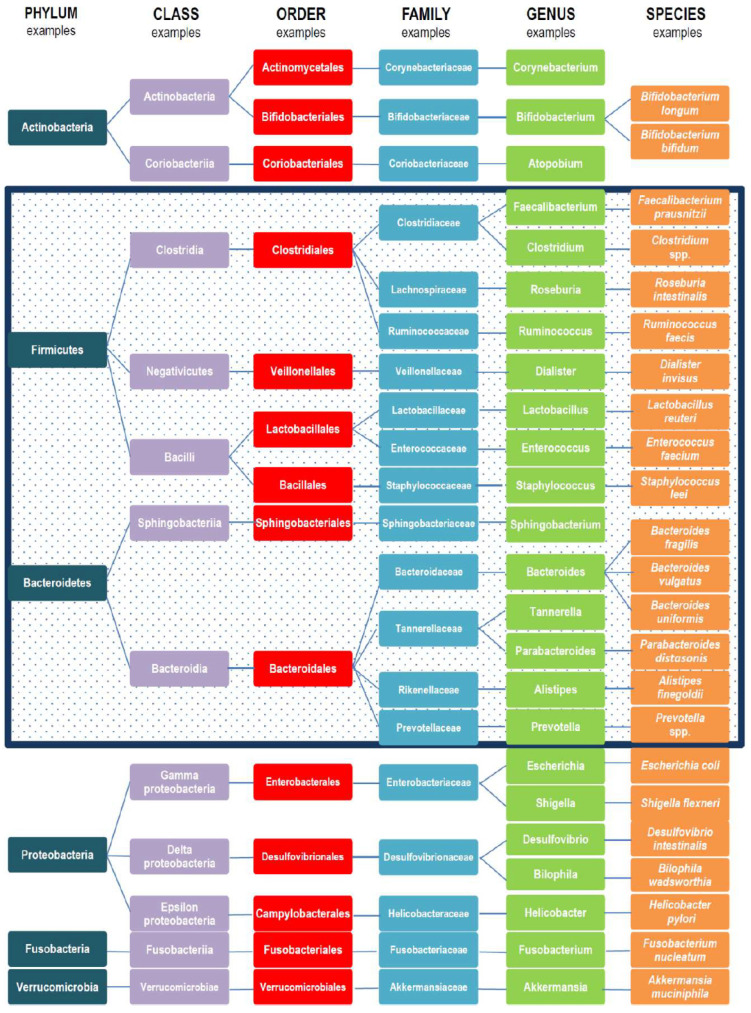
Examples of taxonomic gut microbiota composition. In the box are cited examples of bacteria belonging to Phyla Firmicutes and Bacteroidetes, representing 90% of gut microbiota. Reprinted from Rinninella E, Raoul P, Cintoni M, Franceschi F, Miggiano GAD, Gasbarrini A, Mele MC. Microorganisms 2019, 7(1), 14. Copyright 2019 by the authors; licensee is MDPI, Basel, Switzerland; reprinted with permission under an open access Creative Commons Attribution (CC BY) license (http://creativecommons.org/licenses/by/4.0/, accessed on 20 December 2022). See: https://doi.org/10.3390/microorganisms7010014, accessed on 20 December 2022).

The gut microbiome, including the appendix that is now recognized as a non-vestigial well that maintains the microbiome [72], is recognized today for substantial influences on human health [30,31,32,73,74,75,76]. The microbiome impacts human health, both in the prevention of or acceleration of chronic diseases and in limiting or enhancing infectious diseases [16,30,31,32,73,74,75,76]. This includes the potential for acute infections in the intestine or in the appendix to radically alter organ-level health [73,74,75,76]. SARS-CoV-2 may be one of such infections, inducing gut microbiome dysbiosis that leads to enhanced proliferation of pathogenic microbial species, including anti-microbial resistant strains, and to the movement of harmful secondary pathogens into the bloodstream from the gut [30,73]. In a study of a murine model and, in parallel, of patients with COVID-19, substantial loss of microbiome diversity was observed with many of the human cases having a single taxa that accounted for >50% of the bacteria in their gut and the loss of diversity was associated with the presence of blood stream infections [30]. Further, one member of Firmicutes (i.e., *Faecalbacterium*) that is present in abundance in the healthy human gut (see Figure 1 for an overview of the common composition of the human microbiome) was found to be substantially reduced in prevalence in patients infected by SARS-CoV-2 [30]. While this area is insufficiently studied, the disruption of the microbiome by COVID-19 is undoubtedly profound and the changes that SARS-CoV-2 infection has on near-term health and on chronic disease risk due to its alterations of the microbiome require additional investigation.

To our knowledge, no study has directly evaluated the impact of fasting on the microbiome in people infected by SARS-CoV-2, but an impact in preventing adverse changes can be expected due to other observations in which fasting shapes the microbiome to favor species linked to optimal metabolic health and low risk of obesity. During fasting, gut microbial growth and the production of their byproducts (e.g., trimethylamine N-oxide) are inhibited [16]. In a study of mice, fasting inhibited infection by *Salmonella enterica*, reduced associated gastroenteritis, and decreased pro-inflammatory signaling, but did so variably depending on the existing microbiome [18], also suggesting that during fasting some immediate effects may impact acute infection. In people with hypertension and metabolic syndrome in a sub-analysis of another longer-term study, a 5-day fast with subsequent anti-hypertensive diet reduced blood pressure and body mass, but also modified the persistent composition of the microbiome in part by increasing the abundance of bacteria with reported beneficial impacts on metabolic and inflammatory profiles [31]. Specifically, intermittent fasting modified the abundance of various microbes such as Desulfovibrionaceae, Hydrogenoanaerobacterium, *Akkermansia*, and Ruminococcaceae (Figure 1) [31]. A subsequent study of once-per-week fasting compared to twice-per-week fasting found modest impacts of both fasting regimens on the microbiome, with some minor differences in the microbial diversity that was affected but generally positive results for species that improve metabolic health [32]. In that study, intermittent fasting increased the abundance of species in the Ruminococcaceae and Lachnospiraceae families (Figure 1), while *Eubacterium ventriosum* was decreased in abundance by one form of fasting [32]. A systematic review of two types of intermittent fasting (i.e., time-restricted eating and alternate-day fasting) reported apparent effects of fasting on the microbiome such as modification of the ratio of Firmicutes/Bacteroidetes (Figure 1), with metabolism-protective microbes such as *Lactobacillus* species and *Akkermansia municiphila* increased in abundance by both regimens [76]. In summary, short-term changes occur in the gut microbiome during fasting that alter the risk-associated factors that are produced and may ameliorate acute changes due to active infections such as COVID-19, while long-term changes are also caused by fasting that alter microbiotic diversity and may enhance the host’s ability to stave off poor COVID-19 outcomes by improving metabolic health and reducing the risk of chronic diseases over repeated fasting episodes.

### 2.10. Chronic Diseases and Risk of Poor COVID-19 Outcomes

Finally, (see Table 1 for a summary of the mechanisms), it is now well-established that chronic diseases such as coronary artery disease, myocardial infarction, heart failure, diabetes, and obesity exacerbate COVID-19, leading to severe outcomes [36,37,50]. These diagnoses and adverse events involve a cluster of risk factors including obesity, high visceral fat accumulation, elevated cholesterol and hypertriglyceridemia, insulin resistance, and high blood pressures, all of which are connected to elevated risk of poor outcomes in COVID-19 [36,37,50]. In particular, studies have demonstrated that SARS-CoV-2 infection upregulates glycolysis, alters lipid metabolism, and changes amino acid and nucleotide production, changes that benefit viral replication at the expense of normal cellular function [77]. This may in part explain why people with diabetes and other diseases involving metabolic dysfunction such as obesity, coronary disease, and heart failure are at elevated risk of poor outcomes due to COVID-19.

Risk factors for and the risk of diagnosis of those chronic disease diagnoses are lower among people who engage in intermediate- or long-term intermittent fasting [4,5,6,7,8,9,20,38]. This includes a lower risk of the metabolic syndrome and the cardiometabolic risk factors that lead to the diagnoses [2,3,4,5,6,7,8]. Further, the risk of mortality, hospitalization, and a new diagnosis of heart failure are also lower for people engaging in periodic fasting [10,14]. In particular, fasting reduces glucose levels acutely and substantially during a fasting period and it reduces the basal level of glucose over the long-term [4,6,7,8], which in both cases will make glucose less available for use during infection and, thus, should counteract the stimulation of glycolysis performed by SARS-CoV-2 [77]. Because they are known to reduce the risk of the onset of those chronic diseases, standard weight loss diets should also produce some of these benefits over the long-term, including reduced risk of chronic disease and improved glucose control. From a practical perspective, sustainable intermittent fasting regimens and weight loss diets should be utilized to ensure that people continue with the dietary practices for as long as their health requires it.

## 3. Safety Considerations

Intermittent fasting is a generally safe [78], inexpensive, and well-tolerated dietary energy restriction practice that reduces weight [2,3,4,5], and is effective in improving metabolic and cardiovascular health primarily in people with elevated cardiometabolic risks [4,5,6,7,8]. Safety outcomes have been largely ignored in intermittent fasting studies and some populations are at considerable risk if they participate in fasting, such as people with diabetes and especially children with juvenile or type 1 diabetes [78]. Safety concerns with fasting include mild potential side effects, such as hunger, fatigue, dizziness, constipation, headache, lightheadedness, syncope, and falls that make fasting generally safe for people who are apparently healthy. For some people, though, fasting can cause more serious safety issues, including for people with diagnosed chronic diseases (including people with prior heart attack or stroke and especially those with lingering symptoms of those events). In particular, fasting reduces blood sugar and can result in hypoglycemia. It can also result in dehydration. People with type 2 diabetes should be cautious about fasting and talk with their physician before initiating a fasting regimen because many anti-diabetes medications can also cause dehydration and hypoglycemia; dehydration is a risk factor for stroke and general thromboembolism while hypoglycemia can be fatal [78]. People with pre-diabetes or who are being treated for insulin resistance or metabolic syndrome using anti-diabetic medications should consult with their physician about safe dietary and medication practices before engaging in a fasting regimen [78]. Adults with type 1 diabetes should not fast unless it is prescribed and monitored by a physician [78]. Other safety concerns may include excessive stressors and nutritional deprivations on the body that generally can cause severe adverse events in people who are older and frail and those who are pregnant, lactating, have significant kidney disease, have had an organ transplant, are receiving active cancer treatment, have an eating disorder, are malnourished, have type 1 diabetes, or have dementia. Additionally, people with inflammatory bowel disease, Celiac disease, irritable bowel syndrome, diabetes, and cancer often require a specific diet that may not accommodate introduction of a fasting regimen. Malnutrition in particular should be paid attention to, and individuals with nutrition concerns may exhibit oral markers of this condition that would indicate that someone is not a candidate for fasting [79]. Children also should not fast for health purposes unless it is prescribed by their physician or another medical professional trained in pediatrics, and children with a diagnosis of diabetes (type 1 or 2) or who are being treated for diabetes should not fast. Fasting is not a therapy that provides many benefits without any risks. Unfortunately, no systematic assessment of the risks of fasting across diseased or general populations has been undertaken and, thus, data are sparse on safety issues related to fasting in the populace.

## 4. Conclusions

COVID-19 continues to rage across the globe and the SARS-CoV-2 virus continues to rapidly evolve to evade the human immune system. While simple preventive measures and public health efforts could greatly reduce transmission of the virus, a lack of knowledge of these and barriers to belief in or acceptance of them are contributing to ineffective prevention of COVID-19. Vaccines have emerged as a major preventive treatment for this infectious disease. Medical treatments are available in some settings once COVID-19 is diagnosed (e.g., remdesivir, monoclonal antibodies, dexamethasone, and baricitinib), although these require substantial financial investment and have more side effects than vaccines, including some with an increased recognition of COVID-19 rebound (e.g., nirmatrelvir/ritonavir). Despite these rapidly developed therapeutic advancements, various financial constraints, healthcare access issues, and sociopolitical situations are contributing to limited vaccination rates and antiviral use globally. The continuing mutation of SARS-CoV-2 is also producing more infectious and virulent variants that refuel the pandemic and threaten to invalidate the progress made in fighting COVID-19. This includes that viral mutation is reducing or nullifying the effectiveness of SARS-CoV-2 vaccines and antiviral medications, and will continue to reduce the effectiveness of these new therapies in the future.

An evolving need exists for complementary therapies that support the effectiveness of COVID-19 treatments and that supplement preventive measures. Such approaches could be valuable in locations like the United States where vaccine hesitancy and vaccine rejection are occurring, as well as in other geographies such as Africa where vaccination is uncommon and is inhibited by a lack of funds to acquire vaccines and by other logistical issues. While periodic fasting is reported to be associated with lower risk of severe COVID-19 outcomes, that observational finding was examined among people with a life-long devotion to fasting behavior [14]. In this time of acute need, a more intense and rapid therapeutic need exists that more frequent intermittent fasting may afford for people who have little to no history of fasting.

The available evidence suggests that intermittent fasting, as a more frequent and intense set of regimens than periodic fasting, should improve the immune response of and reduce acute hyperinflammation for unvaccinated people, strengthen immunity between vaccinations for vaccinated people, and prolong the length of time a vaccinated person can go before receiving a subsequent booster dose of the SARS-CoV-2 vaccine. By boosting autophagy, fasting may aid the immune system to identify silently infected cells via increased degradation of viral proteins and through antigen presentation to natural killer cells and cytotoxic T cells. Intermittent fasting may also provide a constellation of mechanisms that empower a damaged human immune system to repair itself and to hunt down residual SARS-CoV-2 virus that is hiding from it in the context of post-acute sequelae of COVID-19, which should be a subject of further investigations. Because periodic fasting may reduce the risk for coronary artery disease [9], diabetes [9], and incident heart failure [10], and increase the likelihood of survival [10], it is also surmised that long-term participation in intermittent fasting over months and years may have similar and likely stronger effects than periodic fasting.

Prior to initiating an intermittent fasting regimen, people should ensure that it will be safe for them. Subsequently, they should utilize a sustainable regimen over a period of years to improve their health and, subsequently, to maintain that improvement. Beginning an intermittent fasting regimen prior to being infected is likely most beneficial for the prevention of severe outcomes of infectious diseases such as COVID-19 since some proposed mechanisms of benefit may be realized over the long-term. During an acute infection, though, a differential fasting intensity would potentially be needed depending on the individual’s personal risk of poor health outcomes. Ultimately, though, very limited experimental human data exist from studies that directly examined the therapeutic or preventive use of intermittent fasting for improvement of COVID-19 outcomes. Because fasting acts as a trigger of physiologic benefits by activating processes that are encoded in the human genome, fasting adds no financial cost to a person’s health choices when practiced safely and is a source of potential health equity for humanity. The overflowing global adversity induced by the COVID-19 pandemic is a clear and present danger that, given the discussion of mechanisms herein, calls for a redirection of attention to clinical outcomes studies evaluating the potential complementary use of intermittent fasting for protection from acute COVID-19 and for the resolution of post-acute sequelae of COVID-19.

## Figures and Tables

**Table 1:** Proposed mechanisms of intermittent fasting in preventing severe COVID-19 outcomes.

Mechanisms of SARS-CoV-2 and COVID-19	Proposed Counteraction of Fasting	References
1. Linoleic acid attaches to a SARS-CoV-2 binding pocket on the spike protein, locking the spike in a conformation that is not conducive to binding to ACE2.	During fasting, fatty acids including the essential fatty acid linoleic acid are increased in the circulation and may reduce SARS-CoV-2 affinity for ACE2.	[15,16,42,43,44,45]
2. SARS-CoV-2 impairs ketosis and the production of β-hydroxybutyrate, leading to a deficit in interferon-γ production by CD4^+^ T cells.	During fasting and ketogenic diets, circulating levels of ketones including β-hydroxybutyrate are increased and may restore CD4^+^ T cell function.	[16,44,45,46,47]
3. SARS-CoV-2 infects human adipocytes and tissue-resident macrophages, inflaming adipose tissue and preferentially harming people with obesity.	During fasting, adipocyte contents are scavenged for energy and inflammation reduced; in the long-term, the basal volume of adipose tissue is decreased by fasting.	[4,5,8,20,37,48,49,50]
4. SARS-CoV-2 infects T cells and preferentially infects activated T cells using a mechanism not involving ACE2 [likely entering T cells via the lymphocyte function-associated antigen 1 (LFA-1)].	During fasting, the T cell response is blunted, some T cells are deactivated, and neutrophil and natural killer cell activity are strengthened.	[21,22,25,26]
5. CD4^+^ T cell activation of the human inflammatory cytokine cascade in response to binding of a pathogen (i.e., SARS-CoV-2) contributes to hyperinflammation in severe COVID-19.	During fasting, the CD4^+^ T cell response is blunted, NF-κB activity and the NLRP3 inflammasome are reduced, and inflammatory cytokines are minimized.	[19,21,22,23,24,25,51,52,53,54]
6. SARS-CoV-2 raises the pH of the lysosome, inhibits autophagosome-lysosome fusion, and blocks other key pathways, effectively inhibiting autophagy of infected cells.	Fasting activates and enhances autophagy, strengthening the ability to degrade damaged proteins and cellular organelles.	[19,25,27,28,29,38,55,56,57,58]
7. A majority of the proteins encoded by SARS-CoV-2 actively block or suppress human production of type I interferon and the activation of related genes, suppressing innate immunity, and they suppress antigen presentation on cell surfaces.	Fasting supports optimal immune function and may aid in cell surface presentation of viral antigens and strengthen innate immunity, speeding the recognition that an infection is present.	[19,57,61,62,63,64,65,66,67,68,69]
8. SARS-CoV-2 attacks the human immune system and causes dysregulation of the inflammatory response to infection.	Repeated fasting increases basal levels of galectin-3, a β-galactoside-binding lectin that responds directly and indirectly to infection and reduces inflammation arising from NF-κB and the NLRP3 inflammasome.	[17,23,33,34,35,70,71]
9. SARS-CoV-2 induces gut microbiome dysbiosis, leading to enhanced proliferation of pathogenic microbial species including anti-microbial resistant strains and to the movement of harmful pathogens into the bloodstream.	During fasting, harmful gut microbes and their byproducts are inhibited. Repeated fasting enhances microbiome diversity and increases the abundance of microbes known to improve metabolic health.	[16,18,30,31,32,73,74,75,76]
10. COVID-19 outcomes are more severe for people with coronary artery disease, myocardial infarction, heart failure, diabetes, and other chronic diseases.	Repeated fasting over the long-term prevents the onset of chronic diseases and decreases the risk of major adverse events.	[4,5,6,7,8,9,10,14,20,36,37,38,50,77]
